# Let-7c regulated epithelial-mesenchymal transition leads to osimertinib resistance in NSCLC cells with EGFR T790M mutations

**DOI:** 10.1038/s41598-020-67908-4

**Published:** 2020-07-08

**Authors:** Xiao-Feng Li, Wei-Zhang Shen, Xin Jin, Ping Ren, Jie Zhang

**Affiliations:** 1 Department of Oncology and Hematology, The Second Hospital of Jilin University, 218 Ziqiang Street, Nanguan District, Changchun, 130041 Jilin People’s Republic of China; 2 Department of Thoracic Surgery, The First Hospital of Jilin University, Chaoyang, Changchun, 130021 Jilin People’s Republic of China; 3 Department of Respiratory Medicine, The Second Hospital of Jilin University, 218 Ziqiang Street, Nanguan District, Changchun, 130041 Jilin People’s Republic of China

**Keywords:** Non-small-cell lung cancer, Non-small-cell lung cancer

## Abstract

Epidermal growth factor receptor- tyrosine kinase inhibitors (EGFR-TKIs) have shown promise against non-small cell lung cancers (NSCLCs) in clinics but the utility is often short-lived because of T790M mutations in EGFR that help evade TKIs’ action. Osimertinib is the third and latest generation TKI that targets EGFRs with T790M mutations. However, there are already reports on acquired resistance against Osimertinib. Recent work has revealed the role that miRNAs, particularly tumor suppressor let-7c, play in the invasiveness and acquired resistance of NSCLCs, but the mechanistic details, particularly in Osimertinib resistance, remain elusive. Using two cells lines, H1975 (endogenous T790M mutation) and HCC827-T790M (with acquired T790M mutation), we found that let-7c is a regulator of EMT, as well as it affects CSC phenotype. In both the cell lines, transfection with pre-let-7c led to reversal of EMT as studied through EMT markers e-cadherin and ZEB1. This resulted in reduced proliferation and invasion. Conversely, reduced expression of let-7c through anti-let-7c transfections significantly increased proliferation and invasion of lung cancer cells. Expression of let-7c was functionally relevant as EMT correlated with resistance to Osimertinib. High let-7c expression reversed EMT and made cells sensitive to Osimertinib, and vice versa. WNT1 and TCF-4 were found to be two targets of let-7c which were epigenetic suppressed by let-7c through increased methylation. In vivo, pre-let-7c inhibited while anti-let-7c potentiated tumor growth and WNT1 and TCF-4 were downregulated in xenografts with pre-let-7c. Silencing of both WNT1 and TCF-4 resulted in potentiation of Osimertinib action. Our results suggest an important role of let-7c in regulating EMT and the resulting Osimertinib resistance in T790M NSCLCs. More clinical studies need to be performed to fully understand the translational relevance of this novel mechanism.

## Introduction

Lung cancer is the most fatal cancer, worldwide^1^. It is particularly lethal in China with mortality rate higher than most countries^2^. Also, the lung cancer mortality in China is projected to substantially increase, upto 40%, by the year 2030, compared to what it was in 2015^3^. Non-small cell lung cancer (NSCLC) is the major subtype of lung cancer accounting for more than 80% of all lung cancers^4^. About a decade and half back, United States Food and Drug Administration approved erlotinib in the second and third line settings for unselected advanced NSCLC patients^5^. Erlotinib is the first generation epidermal growth factor receptor (EGFR) tyrosine kinase inhibitor (TKI) that is effective against NSCLCs with a sensitizing mutation. However, a majority of patients stop responding to first generation EGFR-TKIs in due time^6^. A number of mechanisms are responsible for this acquired resistance^7^, including ones that involve microRNAs (miRNAs)^8–10^. It is estimated that as many as 60% of patients with acquired EGFR-TKI resistance have EGFR T790M mutation^5^ and such patients can benefit from Osimertinib, a third generation EGFR-TKI that targets EGFR with T790M mutations^11^.

Even though Osimertinib emerged as an advanced EGFR-TKI that could be used for treatment of lung cancer patients that had progressed on earlier generation EGFR-TKIs, it has emerged that Osimertinib resistance is also a clinical reality^12,13^. However, Osimertinib is a relatively new therapy with not enough knowledge regarding the resistance mechanisms. Based on the documented role of epigenetics, particularly miRNAs, in TKI-resistance, we focused on such mechanism for Osimertinib resistance as well. Epigenetic regulation of lung cancer progression is also evident in the many reports that have focused on the various miRNAs^8,14–16^. These reports have clearly documented an important role of miRNAs in invasion, proliferation as well as metastasis of lung cancers. It also emerges that let-7c is a critical miRNA affecting lung cancers^17,18^. Let-7c is a tumor suppressor miRNA by virtue of its proactive role in the inhibition of proliferation, migration and invasion of lung cancer cells. It is linked to recurrence of lung cancers^19^. There is no doubt that recurrence of malignancies is lethal and this underlines the importance of let-7c in progression of lung cancers. Although these evidences point to the possibility of let-7c in lung cancer progression and provide preliminary evidence, the more precise mechanism needs to be elucidated. Since a role of let-7c miRNA in EMT of various cancers has been described^8,16,20,21^, we first sought to examine a role of let-7c in EMT of lung cancer cells followed by an evaluation of possible role of let-7c in sensitivity to Osimertinib.

## Methods

### Cells

For our study, we have used H1975 cell line, a T790M-positive cell line that harbors the EGFR L858R/T790M double mutation^22^. This cell line was obtained from ATCC (Manassas, VA, USA). The other cell line used in this study was HCC827-EPR, referred to as HCC827-T790M in this study, which originated at Aichi Cancer Center Hospital, Japan^23^. These cells are resistant clones of HCC827 that acquired T790M mutation when continuously exposed to erlotinib and the MET inhibitor PHA-665,752^22,23^. Both cell lines were cultured in RPMI-1640, supplemented with 10% fetal bovine serum and 1% antibiotics. Cells were maintained and propagated as monolayer cultures at 37 °C in a humidified 5% CO_2_ incubator. Osimertinib was purchased from Selleckchem (USA).

### Cell proliferation (XTT assay)

Cell proliferation kit II (XTT) was purchased from Millipore Sigma (China). It constitutes a colorimetric assay that employs nonradioactive quantification of cellular proliferation, viability, and cytotoxicity. The assay involves cleavage of tetrazolium salts to formazan. The cleavage product of XTT is soluble in water and this bioreduction occurs in viable cells only, and is related to NAD(P)H production through glycolysis. Cells were seeded in 96-well tissue culture plate in a total volume of 100 μl and, at the end of experiment, incubated with 50 μl XTT labeling mixture (final XTT concentration 0.3 mg/ml) for 2 h. The formazan dye was quantitated using an ELISA reader (Shimadzu, Japan).

### Cell invasion assay

Cell invasion was assayed using QCM ECMatrix Cell Invasion Assay, 24-well (8 µm) (Millipore Sigma, China) manufactured by Chemicon. The kit utilizes ECMatrix, a reconstituted basement membrane matrix of proteins derived from the Engelbreth Holm-Swarm (EHS) mouse tumor. Cells were seeded over the insert and complete medium was provided in the lower chamber to ensure migration. The inserts contained 8 μm pore size polycarbonate membrane, over which a thin layer of ECMatrixTM was dried. The ECM layer blocks non-invasive cells from migrating through. Invasive cells, on the other hand, migrate through the ECM layer and cling to the bottom of the polycarbonate membrane. Detection of invading cells was done via crystal violet stain.

### Quantitative real-time PCR

Total cellular RNA was extracted using Trizol Reagent (Life Technology, Carlsbad, CA, USA). Real-time quantitative reverse transcriptase polymerase chain reaction was performed using ABI Prism Sequence Detection System (Perkin-Elmer Applied Biosystems, Waltham, MA, USA) to detect let-7c expression; SYBR green was used to detect PCR products. Primers for all genes and loading control genes were purchased from Ambion (Austin, TX, USA). All reactions were performed in triplicate using a 25 μl reaction volume, as previously described. Expression levels were calculated and presented following the 2^−ΔΔCt^ method (ΔΔCt = ΔCt (positive)-ΔCt (control)).

### DNA extraction and treatment with sodium bisulfite

We used the method of Liu et al.^24^ for DNA extraction and sodium bisulfite treatment, without any modifications. We isolated genomic DNA using a TIANamp Genomic DNA kit (Tiangen, Beijing, China) and checked for purity of DNA using NanoDrop 1,000 Spectrophotometer (Thermo Fisher Scientific, Shanghai, China). 2 µg of DNA was denatured (in 3 M NaOH for half hour at 42 °C), followed by incubation with freshly prepared 2.5 M sodium bisulfite and 1 M hydroquinone in a total volume of 520 µl at 55 °C for 16 h. Purification of DNA was done using Wizard DNA Clean-Up System (Promega, Madison, WI, USA). The reaction was terminated by the addition of 5.5 µl NaOH (3 M) at room temperature for 15 min. Sample was precipitated by the addition of 33 µl ammonium acetate (10 M, pH 7.0), 270 µl ethanol and 4.0 µl glycogen (10 µg/µl), at − 20 °C for 12 h. The modified DNA was resuspended in 30 µl elution buffer and stored at − 20 °C.

### Methylation-specific-PCR

Sodium bisulfite-converted DNA (50 ng) was amplified and the PCR performed on a BioRad instrument. The cycling conditions were: 38 cycles of denaturation at 94 °C for 30 s, annealing at 60 °C (unmethylated) or 62 °C (methylated) for 30 s and extension at 72 °C for 1 min. Regardless of whether the unmethylated allele was amplified, positivity was determined as a sample with a methylated allele. All other samples were classified as negative.

### Animal experimentation

The in vivo experiments were reviewed and approved by the Animal Research Ethics Committee at the Jilin University. All methods were performed in accordance with the relevant guidelines and regulations. Male SCID mice were purchased from Vital River Laboratories, Co., China and housed in sterilized animal facility. H1975 cells or HCC827-T790M cells (either control or transfected with pre-/anti-let-7c) were injected subcutaneously in the two flanks (0.8 × 10^6^ per flank) of each mouse (n = 10/group). Thereafter, mice were continuously monitored for any possible adverse events and the size of the developing tumor. Volume of tumors was calculated by using the formula V = (W^2^ × L)/2 where V is tumor volume, W is tumor width and L is tumor length. Mice in all groups were sacrificed when the mean tumor volume in the most aggressive group approached 1,500 mm^3^.

### Statistical considerations

Experiments were performed in triplicates. Two-tailed independent Student’s *t* test and ANOVA were used to compare groups. Graphpad Prism software was used for statistical analyses and P values < 0.05 were considered statistically significant.

## Results

### Let-7c affects EMT and CSC phenotype of lung cancer cells

**Figure 1:**
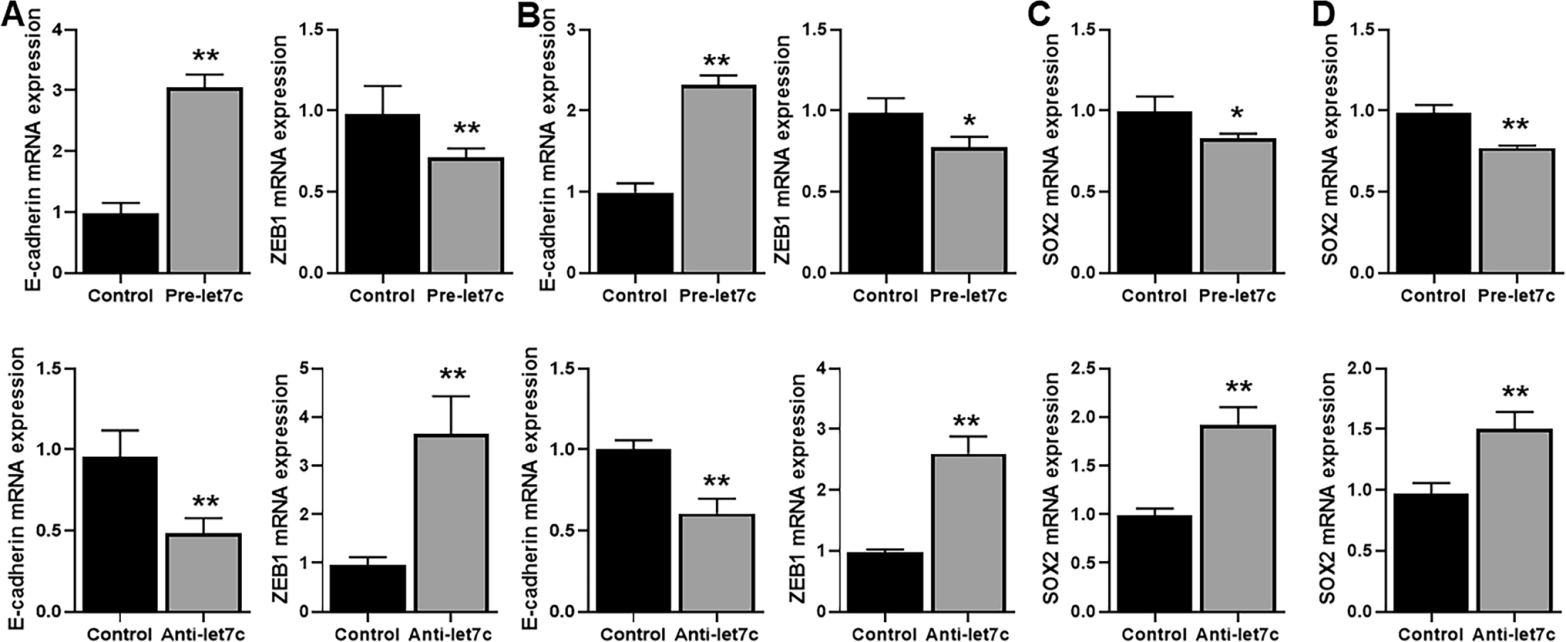
Effect of let-7c on EMT and CSC markers. (**A**) H1975 cells or (**B**) HCC827-T790M cells were transfected with non-specific (control) or pre-let-7c (upper panels) or anti-let-7c (lower panels) and the mRNA expression of EMT markers (E-cadherin and ZEB1) tested by quantitative RT-PCR. (**C**) H1975 cells or (**D**) HCC827-T790M cells were also transfected with non-specific (control) or pre-let-7c (upper panels) or anti-let-7c (lower panels) and the mRNA expression of CSC marker SOX2 tested by quantitative RT-PCR. β-actin served as internal control. *p < 0.05 and **p < 0.01, relative to control.

Even though a role of let-7c in EMT of lung cancers has been suggested, there is absolutely no information on the role of let-7c in Osimertinib resistance of NSCLC which could also involve regulation of EMT as a mechanism. Therefore, we first checked for EMT markers E-cadherin and ZEB1in cells that had either normal or up-regulated/down-regulated let-7c. We found that transfections of pre-let-7c in both the cell lines with EGFR-T790M mutations led to significant increase (p < 0.01) in E-cadherin and significant decrease (p < 0.05) in ZEB1 (Fig. 1A, B). In the reverse case, i.e. when cells were transfected with anti-let-7c, a significant decrease (p < 0.01) in E-cadherin and significant increase (p < 0.01) in ZEB1 (Fig. 1A, B) was observed. Thus let-7c is a modulator of EMT in lung cancer cells, even those with T790M TKI resistance mutation. EMT and cancer stem cell (CSC) phenotype are closely related, therefore, we also checked the effect of let-7c on CSC phenotype by assessing CSC marker, SOX2. Pre-let-7c significantly decreased SOX2 (Fig. 1C, D) in both the cell lines while anti-let-7c significantly (p < 0.01) increased SOX2 (Fig. 1C, D) in both the cell lines.

### Let-7c affects proliferation and invasion of lung cancer cells

**Figure 2:**
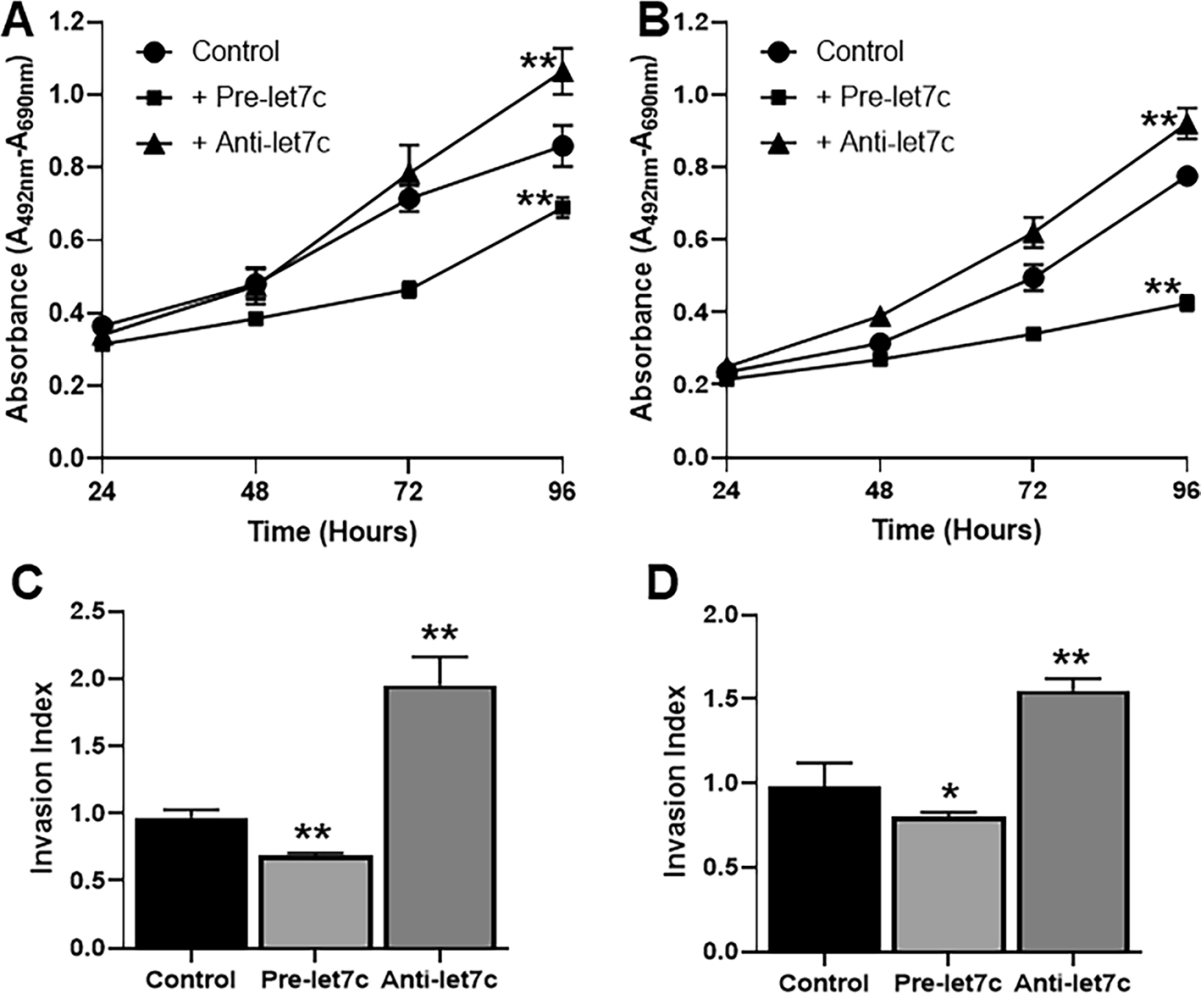
Effect of let-7c on proliferation and invasion. (**A**, **C**) H1975 cells or (**B**, **D**) HCC827-T790M cells were transfected with non-specific (control) or pre-let-7c or anti-let-7c and the effect on (**A**, **B**) proliferation and (**C**, **D**) invasion assessed. *p < 0.05 and **p < 0.01, relative to control.

Proliferation and invasion are two important assessments that describe the aggressive behavior of cancer cells. We tested the effects of let-7c on these two parameters in NSCLC cells with T790M mutations. When checked for proliferation, pre-let-7c decreased cell proliferation and anti-let-7c increased proliferation (Fig. 2A, B). While proliferation is an important parameter, it is invasion that is more related with metastasis and the poor outcome of clinical patients. When checked for the invasive potential of cells transfected with either pre or ant-let7c, we found that pre-let-7c significantly decreased invasion and anti-let-7c significantly increased invasion (Fig. 2C, D).

### Let-7c affects response to Osimertinib

**Figure 3:**
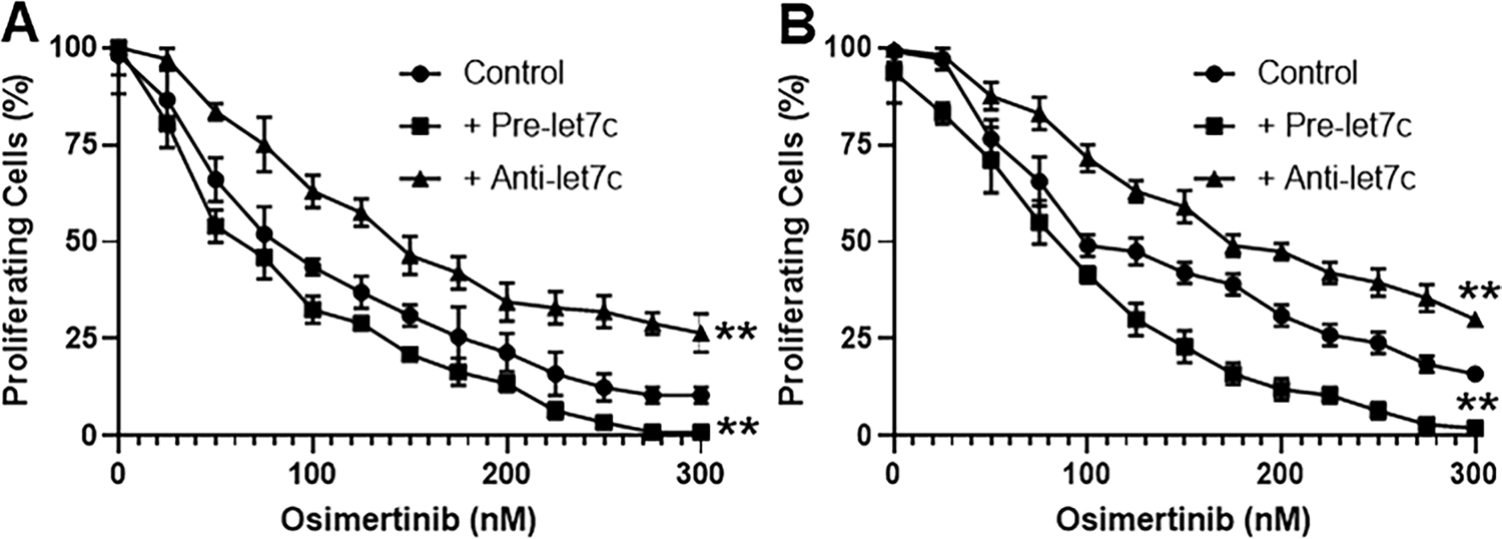
Effect of let-7c on response to Osimertinib. (**A**) H1975 cells or (**B**) HCC827-T790M cells were transfected with non-specific (control) or pre-let-7c or anti-let-7c and then treated with increasing doses, as indicated, of Osimertinib for 72 h, followed by determination of proliferation of cells by XTT assay. Cells at 0 nM Osimertinib were considered to be 100% and relative % of cells are plotted. **p < 0.01, relative to control.

Osimertinib is the third generation EGFR-TKI used for patients who have relapsed on first/second generation EGFR-TKIs because of the resistance related EGFR-T90M mutation. Our results so far indicated a tumor suppressive role of let-7c against NSCLC cells with T790M mutations. We checked whether the inhibitory role of let-7c against proliferation, invasion etc. could also determine the sensitivity against Osimertinib. Both H1975 and HCC827-T790M cells were subjected to different doses of Osimertinib for 3 days and at the end of this time period, XTT assay was performed to quantitate the effect of overexpression/inhibition of let-7c on response to Osimertinib, compared to cells transfected with scrambled control miRNA. It was observed that pre-let-7c further potentiated the effect of Osimertinib and cell proliferation was reduced (Fig. 3A, B). At the same time, anti-let-7c increased the proliferation of both the cell lines (Fig. 3A, B). The IC50 values for both the cell lines and for both pre- as well as anti-let7c conditions were significantly different (p < 0.001) than the control cells (Table 1).

**Table 1:** IC50 values.

	H1975 cells	HCC827-T790M
Control	83.4 ± 1.2	97.2 ± 1.6
+ Pre-Let-7c	64.8 ± 0.9**	82.1 ± 1.8**
+ Anti-Let-7c	147.9 ± 2.0**	171.6 ± 3.4**

All the IC50 values were calculated from the data presented in Fig. 3. Details are provided in the leegend to Fig. 3 and Methods. **p < 0.01, relative to control.

### Targets of let-7c

**Figure 4:**
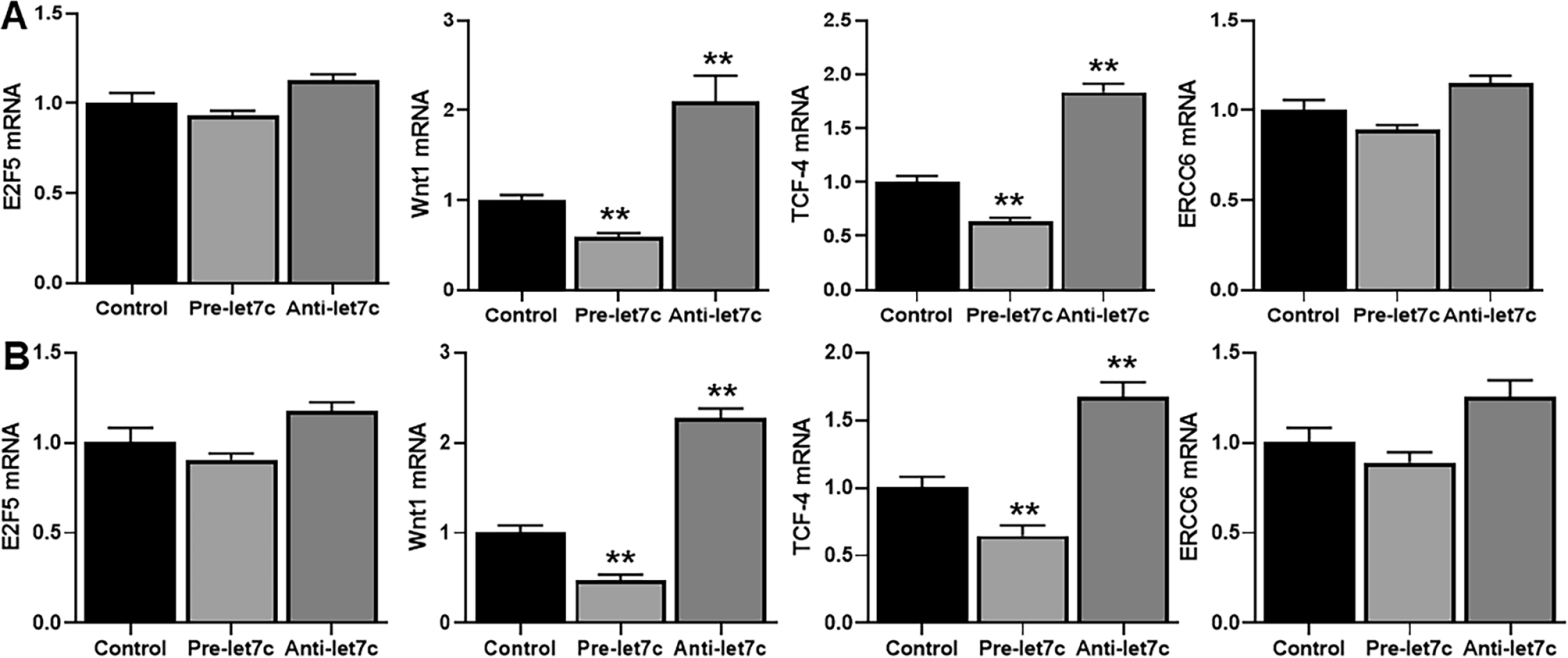
Let-7c targets. (**A**) H1975 cells or (**B**) HCC827-T790M cells were transfected with non-specific (control) or pre-let-7c or anti-let-7c and the mRNA expression of putative target genes (identified on Y-axis) was tested by quantitative RT-PCR. β-actin served as internal control. **p < 0.01, relative to control.

miRNAs function through modulating their target genes. A number of target genes of let-7c have been described in literature and we tested the effects of overexpression and inhibition of let-7c on four such targets, viz, E2F5, WNT1, TCF-4 and ERCC6 (Fig. 4B). There was no significant effect of pre-let-7c or anti-let-7c on E2F5 and ERCC6. However, such effect on WNT1 and TCF-4 was significant. Pre-let-7c significantly decreased (p < 0.01) WNT1 as well as TCF-4 in both the cells lines (H1975 and HCC827-T790M) while anti-let-7c significantly increased (p < 0.01) WNT1 as well as TCF-4 (Fig. 4B).

### let-7c targets affect response to Osimertinib

**Figure 5:**
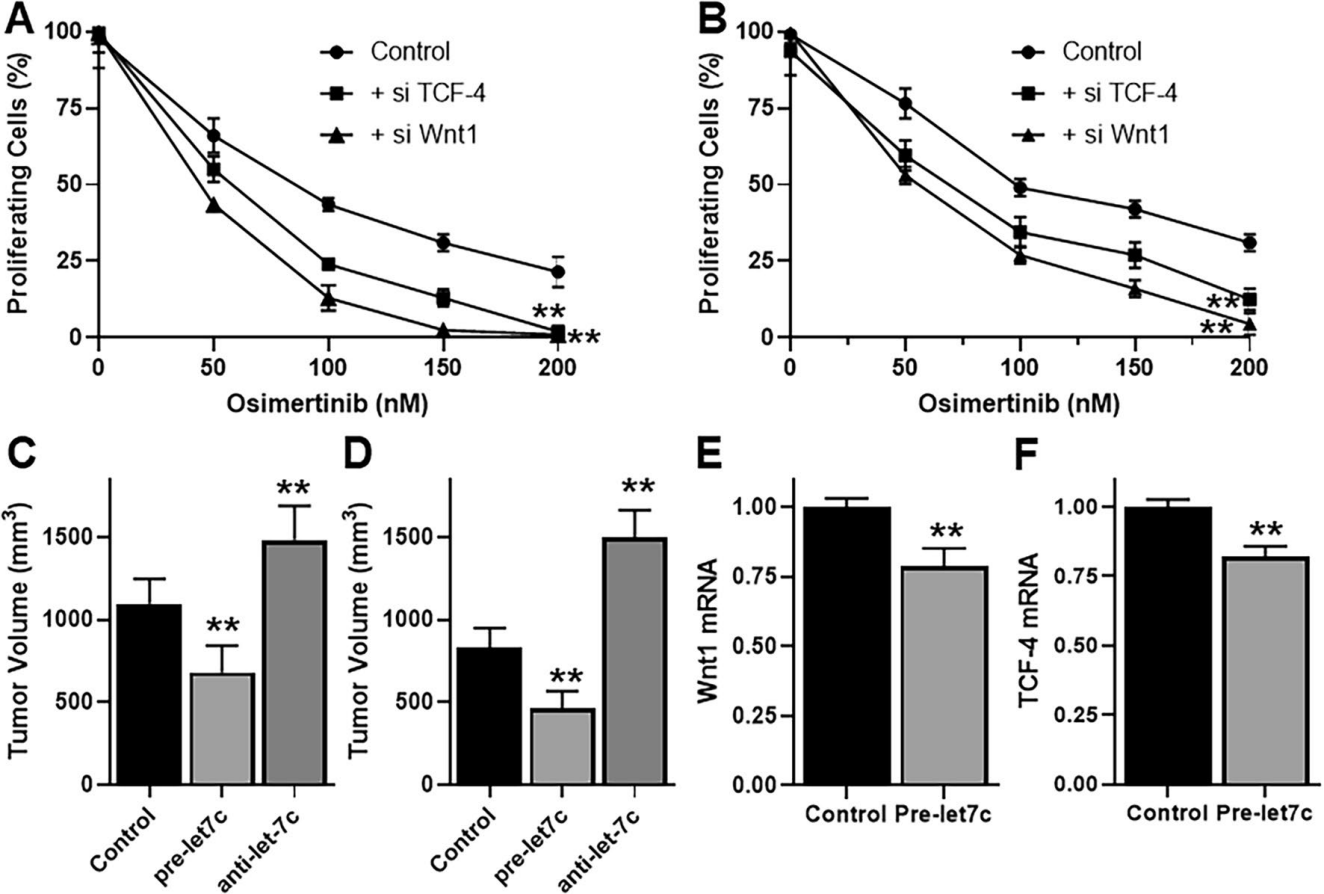
Let-7c targets affect response to Osimertinib and in vivo effects of let-7c. (**A**) H1975 cells or (**B**) HCC827-T790M cells were silenced for WNT1 or TCF-4, using specific siRNAs, and then treated with various indicated doses of Osimertinib for 72 h. Thereafter, proliferation of cells was tested by XTT assay. (**C**) H1975 cells or (**D**) HCC827-T790M cells (control or transfected with either pre- or anti-let-7c) were implanted in SCID mice (n = 10 pergroup) for in vivo xenograft study and the tumor volume was calculated, as described in the Methods. Levels of let-7c targets, wnt1 (**E**) and TCF-4 (**F**) were evaluated in the tumors from control vs pre-let-7c groups of H1975 xenografts. **p < 0.01, relative to control.

Let-7c affects Osimertinib sensitivity, as shown above. Further, it targets WNT1 and TCF-4. Based on these observations, we next checked if silencing of WNT1 and TCF-4 was relevant to Osimertinib sensitivity. We silenced WNT1 and TCF-4 by using their specific siRNAs and evaluated Osimertinib dose–response. We observed that when either WNT1 or TCF-4 (Fig. 5A, B) were silenced, in both cases, the cytotoxic effects of Osimertinib were potentiated. Further, this effect was similar in both the cell lines tested. Since E2F5 and ERCC6 were not affected by let-7c significantly, we did not silence these genes for further testing.

For partial validation of our findings, we performed in vivo experiments wherein we subcutaneously implanted H1975 cells or HCC827-T790M in SCID mice. We aimed to study if levels of let-7c could affect tumor progression. As shown in Fig. 5C, D, pre-let-7c inhibited tumor growth whereas anti-let-7c promoted tumor growth in xenografts from either cell line. We also checked if let-7c functioned in vivo through targeting WNT1 and TCF-4, similar to its in vitro effects. Tumors from H1975 xenograft groups were evaluated as proof of principle and we observed that both WNT (Fig. 5E) and TCF-4 (Fig. 5F) were inhibited in pre-let-7c groups, compared to scrambled controls, thus verifying the in vitro findings.

### Let-7c epigenetically regulates its target genes

**Figure 6:**
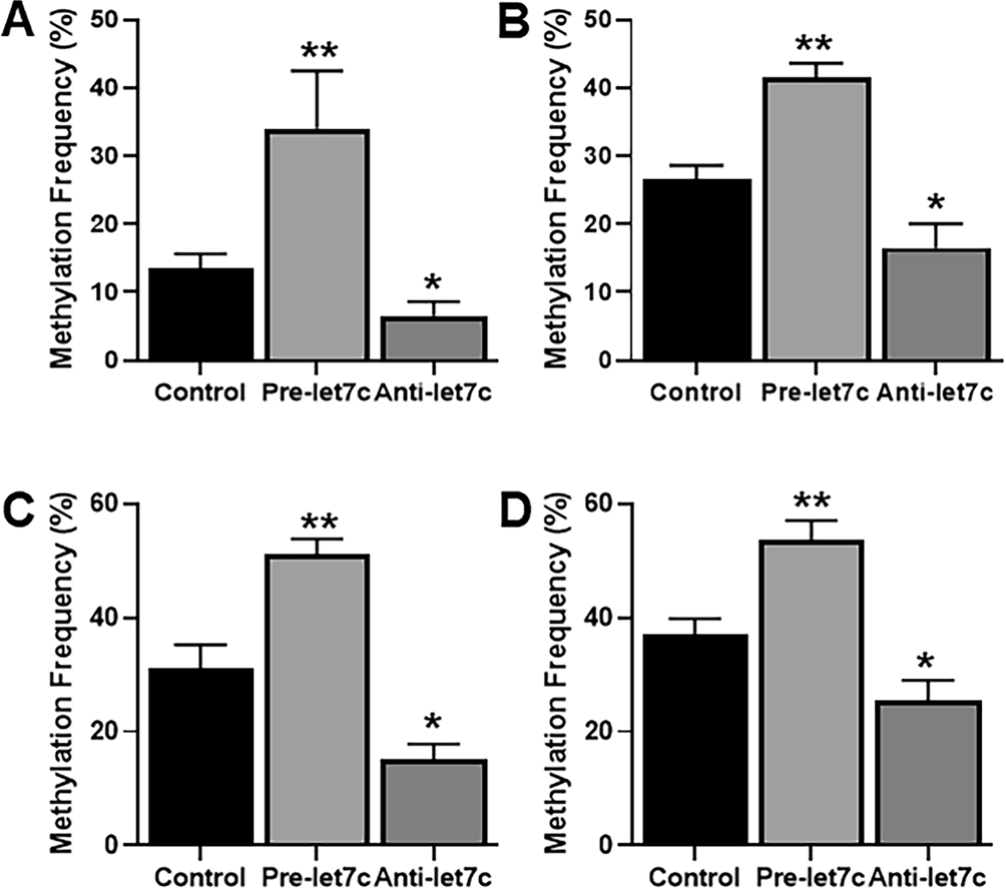
Effect of let-7c on methylation on target genes. (**A**, **B**) H1975 cells or (**C**, **D**) HCC827-T790M cells were transfected with non-specific (control) or pre-let-7c or anti-let-7c and the DNA isolated from cells was subjected to bisulfite treatment followed by PCR for methylated vs non-methylated regions before relative quantification for the genes (**A**, **C**) WNT1 and (**B**, **D**) TCF-4. *p < 0.05 and **p < 0.01, relative to control.

As reported above, WNT1 and TCF-4 were found to be targets of let-7c as their expression was inhibited by let-7c. We checked if there was some epigenetic mechanism involved. When methylation was evaluated, we found that pre-let-7c significantly increased (p < 0.01) the methylation of promoter regions of both WNT1 (Fig. 6A) and TCF-4 (Fig. 6B) genes in H1975 cells. Pre-let-7c significantly increased (p < 0.01) the methylation of promoter regions of both genes (Fig. 6C–D) in HCC827-T790M cells as well. Also, anti-let-7c significantly decreased (p < 0.05) the methylation of promoter regions of WNT1 (Fig. 6A–C) and TCF-4 (Fig. 6B–D) genes in both of the cell lines.

## Discussion

NSCLCs have benefited from EGFR-targeting therapies. However, T790M mutations in EGFR let NSCLCs escape the action of EGFR-TKIs of first and second generation which has led to development of third generation EGFR-TKI Osimertinib. Further, there are indications that treatment with Osimertinib can lead to acquired resistance against this rather new drug as well. Thus, there is urgent need to further understand the mechanism of resistance so that new therapies can be developed. In particular, there is need to evaluate novel markers, such as miRNAs, that can potentially be targeted for therapy. Recent data have shown a lot of interest in evaluating miRNAs in the progression of lung cancers^25–27^. For our study, we focused on let-7c because of its well-known role in resistance to therapies but almost complete lack of knowledge on Osimertinib resistance in lung cancers. There is also interest in this particular miRNA because of its involvement in the phenomenon of EMT that is responsible for cancer metastasis, relapse and resistance to therapies^16^.

In our study we evaluated E-cadherin and ZEB1 as markers to study EMT. E-cadherin is well known epithelial marker while ZEB1 is a well known mesenchymal marker. Tumor cells that are undergoing EMT are characterized by loss of epithelial markers such as E-cadherin and/or gain/increased expression of mesenchymal markers such as ZEB1. In our results, we consistently found that let-7c overexpression, through pre-let-7c transfections, resulted in increased E-cadherin and reduced ZEB1. This is indicative of reversal of EMT. On the other hand, we consistently found that let-7c inhibition, through anti-let-7c transfections, resulted in decreased E-cadherin and increased ZEB1. Further, the mechanism was observed in both of the NSCLC cell lines we used. Thus, let-7c inversely correlates with EMT in our cells and is a tumor suppressor in lung cancer, which in line with the reported tumor suppressive function of let-7c in literature^28,29^.

After characterizing a role of let-7c in reversing EMT in lung cancer cells, we further characterized the effect of let-7c on CSC phenotype. EMT and CSC phenotypes are well known to be connected^30^. This is even true for lung cancer cells^31^. The relationship between EMT and CSC is mostly proportional. Therefore, based on our observation that let-7c reverses EMT, we expected let-7c to have similar inhibitory effect on CSC phenotype as well. Indeed, this was found to be true and overexpression of let-7c reduced CSC marker SOX2 while inhibition of let-7c increased SOX2. These results make it clear that let-7c not only reverses EMT, such effects of let-7c are evident on CSC phenotype as well. Further, experimental assays verified such effects on proliferation and invasion of lung cancer cells as well.

The results where we describe effects of let-7c on Osimertinib-mediated cell proliferation are important from the perspective of clinical management of lung cancer patients. Osimertinib has a special role to play in clinics against lung cancers, particularly with mutated EGFR that confers resistance. However, often there is resistance against Osimertinib. Thus, there is an urgent need to overcome therapy in lung cancers, especially the acquired resistance against Osimertinib. Our results show that let-7c can affect the response of tumor cells against Osimertinib. Consistent with its tumor suppressive activity, let-7c enhances cytotoxic action of Osimertinib and its inhibition leads to increased resistance against Osimertinib. Thus, therapies affecting let-7c can have profound effect on acquired resistance against Osimertinib in NSCLC patients with progressed disease.

In our model systems i.e. NSCLC cells with T790M mutated EGFR, we found that WNT1 and TCF-4 were the true targets of let-7c as they were most affected by let-7c transfections. The shortlisting of four target genes tested by us was based on published reports. WNT1 was shown to be affected by let-7c in breast cancer^32,33^ and that seems to be the case in NSCLC cells as well. ERCC6 has also been reported as a target of let-7c in breast cancer^34^ but we did not find it to be affected by let-7c expressions in our lung cancer cells.

Finally, epigenetics is the emerging field in cancer research^35^ and consists of several different types of modifications that affect gene expression and function. Here, we report that let-7c could differentially methylate its target genes. Increased methylation of its target genes, upon transfection with pre-let-7c, is a clear indication that its methylation that results in suppression of its target genes, as observed in independent experiments. It is well known that methylation of genes results in their suppression^36^. Our study is the first study documenting such epigenetic activity of let-7c, which we believe needs to be further evaluated so that targeting of let-7c could become a clinical reality in the management of lung cancer patients.

## Data Availability

All the data is described within the manuscript.
